# Analysis on short-term change of macular function and the correlates after intravitreal conbercept for CRVO-ME

**DOI:** 10.1186/s12886-022-02381-3

**Published:** 2022-04-08

**Authors:** Yun-Chang Wang, Rong-Rong Li, Yi Cai, Zi-Yi Wei, Chang-Liang Shao

**Affiliations:** The Key Laboratory of Ocular of Heibei Province, The Treatment Centre of Ocular Disease of Hebei Province, Heibei Eye Hospital, Number 399 quan bei dong da jie, Xingtai, 054001 Hebei Province China

**Keywords:** Non-ischemia central retinal vein occlusion, Macular edema, Conbercept, Optical coherence tomography, Multifocal electroretinogram

## Abstract

**Aim:**

To study the short-term change of macular function and the correlates after intravitreal conbercept for CRVO-ME.

**Study design:**

Prospective, clinical study.

**Methods:**

Twenty Three patients(23 eyes) were recruited, who were non-ischemia central retinal vein occlusion diagnosed by FFA (fundus fluorescein angiography) and treated with intravitreal conbercept for macular edema, best – corrected visual acuity ( BCVA), central macular thickness(CMT), amplitude density of P1 wave and implicit time of P1,N1 wave from ring 1 and ring 2 of mf-ERG were measured before and 1 week、2 month after treatment.

**Results:**

Compared to the baseline, BCVA、CMT、amplitude density of P1 wave and implicit time of P1,N1 wave from ring 1 and ring 2 were greatly improved at 1 W、2 M after treatment; better results were gained at 2 M compared to 1 W; Pearson correlation analysis shows no significantly correlation between the improvement of mf-ERG with the change of BCVA、CMT.

**Conclusion:**

The BCVA、the structure and the function of macular were greatly improved after intravitreal conbercept for central retinal vein occlusion induced macular edema; however no significantly correlation between the improvement of the function of macular with the strcture of macular and BCVA.

## Introduction

Central retinal vein occlusion(CRVO) is a common disorder of retinal circulation, which is related to age, systemic diseases, retinal hemodynamic abnormalities, etc. It can be divided into the non-ischemic type and ischemic type according to the area and distribution of capillary non- perfusion zone. Macular edema is one of the main factors affecting the visual prognosis. Anti-VEGF has become the front-line treatment of CRVO-ME [1], which can reduce vascular permeability and increase the absorption of fluid intra or sub-retina, thus the patients, BCVA improved.Optical coherence tomography (OCT) is used to measure the changes of fluid sub-retina of the macular and quantitatively evaluate the morphological recovery of the macular. Multi-focal electroretinography (MF-ERG) is a retinal function related to the activity of cone cells of the posterior under the state of light adaptation. It applied a pseudo-random binary m-sequence cycle to control, the small areas of the stimulation field are alternately overlapped to flash or graphic stimulation. As an objective examination, it can quantitatively analyze the electrical activity of the posterior pole at 30°, especially macular fovea cells [2]. This paper discusses the changes of the macular function and its correlation with BCVA, CMT improvement within a short time after anti-VEGF, as well as investigates the toxicity of conbercept.

## Subjects and methods

### Participants

From August to November 2020, 23 patients (23 eyes) were diagnosed as CRVO-ME, including 10 males and 13 females, aged 52–71 years (average 62.48 ± 5.50). Inclusion criteria: (1) All patients underwent complete examination including BCVA, intraocular pressure, slit-lamp biomicroscopy and fundus pre-set lens; (2) Non-ischemic central retinal vein occlusion diagnosed by FFA (Fundus fluorescein angiography) and macular edema diagnosed by OCT; (3) The enrolled patients voluntarily participated in this trial, signed informed consent and completed treatment and follow-up; (4) The intraocular pressure was in the normal range (10-21 mmHg). Exclusion criteria: (1) Those with a previous history of intraocular surgery, retinal laser photocoagulation, intravitreal drug injection; Ischemic central retinal vein occlusion, age-related macular degeneration, diabetic retinopathy, idiopathic macular hole, epiretinal membrane, and other severe refracting opacity affected examinees. Patients and their families signed informed consent, and this study was approved by the Medical Ethics Committee of Hebei Eye Hospital.

### Methods

#### Procedures

All patients enrolled were given a single intravitreal injection of conbercept. According to the requirements of routine intraocular surgery, after three times of anesthesia with 20 g/L pramipecace hydrochloride eyedrops, the conjunctival sac was flushed with 50 g/L povidone iodine and normal saline, The injections were performed in the operating theater with a sharp 30-gauge needle. The needle was inserted into the eye through the pars plana (3.5-4 mm posterior from the limbus), 0.05 ml of solution containing 0.5 mg of conbercept was injected. After the operation, levofloxacin eyedrops were used four times /day for one week.

#### Measurement indexes

Patients were examined with BCVA, OCT, mf-ERG at baseline,one week and two months after the operation. The best corrected visual acuity was documented in Snellen VA, which was converted into logMAR VA for statistical analysis. The thickness of the fovea was measured by Spectralis OCT in Heidelberg, Germany. According to ISCEV [3] (International Society for Clinical Electrophysiology of Vision) standard, Germany Roland RETI-Port/Scan 21 multifocal visual electrophysiology examination system was used. Sixty-one stimulation units were selected to stimulate the 30° area of the posterior pole alternately and repeatedly, and the implicit times of N1 and P1 in the first ring and the second ring and the amplitude density of the P1 wave were recorded. Follow-up was performed up to two months after the operation, complications such as abnormal intraocular pressure, endophthalmitis, vitreous hemorrhage recorded.

#### Statistical analysis

All statistical analyses were carried out using SPSS 23.0 (SPSS, Inc., Chicago, IL, USA), Quantitative data were presented as mean ± SD. One-way ANOVA was used to compare the data,LSD*-t* test was used to compare the two parametric data between each group, and Pearson correlation analysis was used to analyze the correlation between the improvement of P1 wave amplitude density and the changes of BCVA and CMT: |*r*|= 0, indicating that there is no correlation between the two variables, extremely weak correlation or no correlation for 0 <|*r*|< 0.2, the weak correlation for 0.2 <|*r*|< 0.4, the moderate correlation for 0.4 <|*r*|< 0.6, the strong correlation for 0.6 <|*r*|< 0.8, the extremely strong correlation for 0.8 <|*r*|< 1.0. A *P* value < 0.05 was considered statistically significant.

## Results

**Fig. 1:**
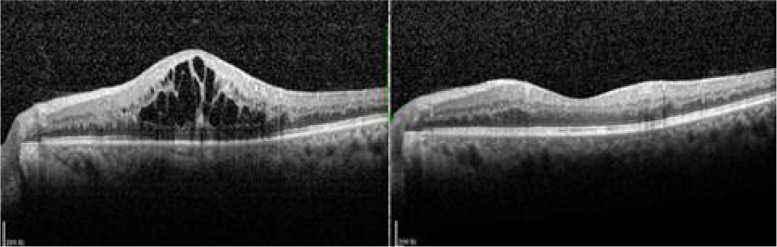
Baseline multiple low refections of cystoid can be seen in fovea,particle-like strong reflection signal can be seen intra-retina, the ellipsoid zone was broken with continuity.the same patient two months after treatment, the fovea was greatly improved

**Fig. 2:**
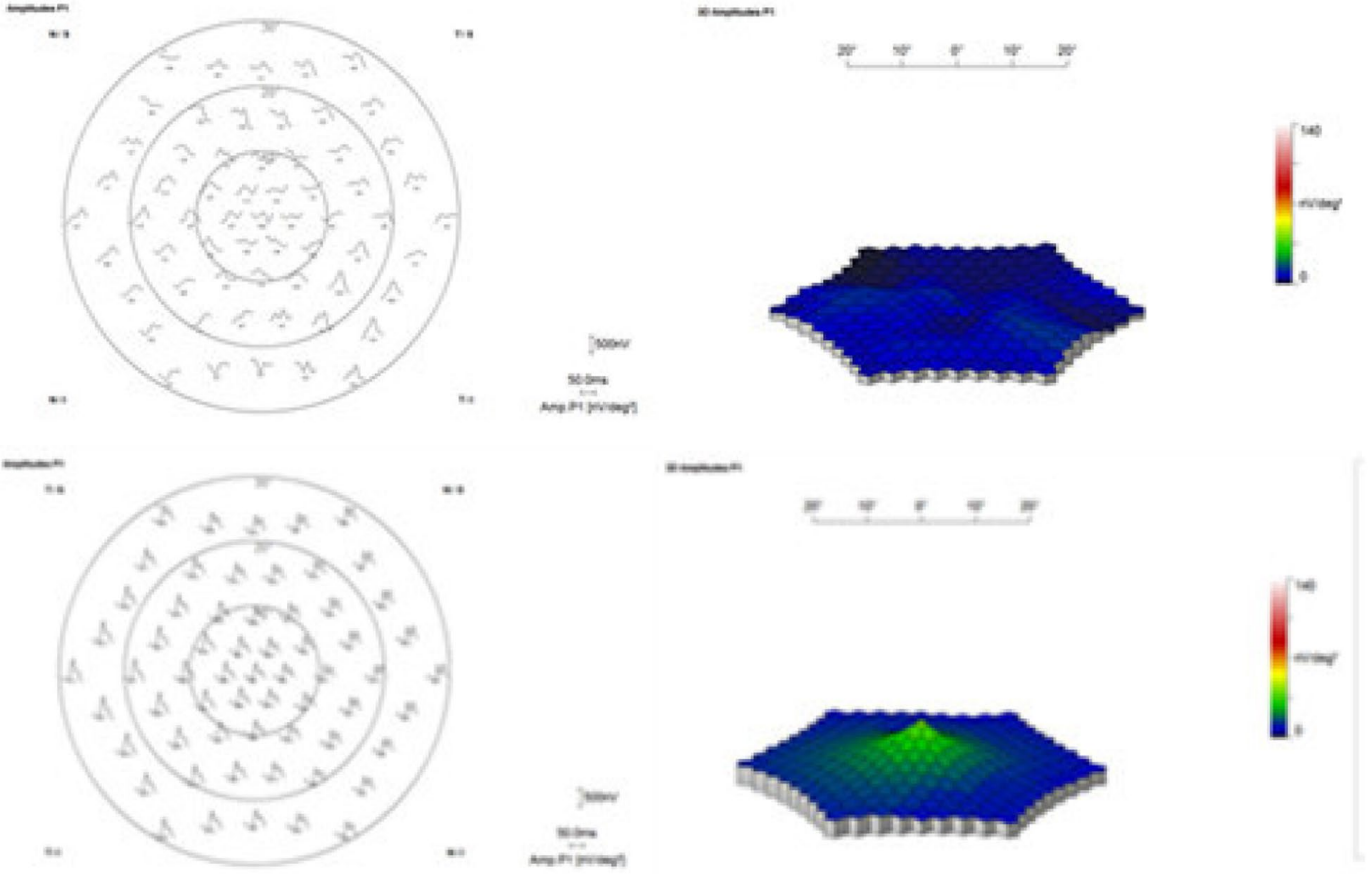
Multifocal electroretinogram of the same patient before and two months after the treatment

All the indexes of the patients in this study were significantly improved at one week and two months after treatment (*P* < 0.05), which are presented in Table 1. BCVA and CMT were improved after treatment(baseline 0.873 ± 0.265,2 M after treatment 0.512 ± 0.141 for BCVA, *P* ≤ 0.001 and baseline 476.183 ± 115.653 μm,2 M after treatment 245.826 ± 20.875 μm for CMT, *P* ≤ 0.001). The implicit time of N1 P1wave from ring 1 and amplitude density of P1 wave from ring 1 were better than baseline(baseline implicit time of N1 wave 31.685 ± 2.799 ms, 2 M after treatment 24.227 ± 1.300 ms for ring1, *P* ≤ 0.001, baseline implicit time of P1 wave 50.523 ± 3.803 ms, 2 M after treatment 43.587 ± 3.692 ms for ring1, *P* ≤ 0.001, baseline amplitude density of P1 wave 15.809 ± 5.420 nv/deg^2^, 2 M after treatment 27.417 ± 6.010 nv/deg^2^for ring1, *P* ≤ 0.001). The implicit time of N1 P1wave from ring 2 and amplitude density of P1 wave from ring 2 were gained better results than baseline(baseline implicit time of N1 wave 29.888 ± 3.044 ms, 2 M after treatment 22.547 ± 2.453 ms for ring2, *P* ≤ 0.001, baseline implicit time of P1 wave 48.414 ± 3.684 ms, 2 M after treatment 39.094 ± 2.767 ms for ring2, *P* ≤ 0.001, baseline amplitude density of P1 wave 4.004 ± 1.467nv/deg^2^, 2 M after treatment 10.671 ± 2.006 nv/deg^2^for ring2, *P* ≤ 0.001)**.** The results showed that the macular morphology and function were significantly improved in a short time after anti-VEGF. Pearson correlation analysis: the correlation coefficient between the increase of amplitude density of P1 wave and BCVA change is *r* = -0.079, *P* = 0.721, the correlation coefficient between the increase of amplitude density of P1 wave and the improvement of CMT is *r* = 0.282, *P* = 0.192. The results showed that there was no correlation between the improvement of the function in the fovea and within 10° with the changes of BCVA and CMT. There were cystoid changes and particle-like strong reflection signal shown before treatment,however the morphology of fovea of the same patient was greatly improved two months after treatment, which was shown in Fig. 1. We could also detect the great improvement in amplitude density of macular of the same patient two months after treatment from Fig. 2.

**Table 1:** Comparison of parameters before and after treatment $\overline{x }\pm s$

index	prior treatment	post treatment 1 W	post treatment 2 M	*F, P*	*t*_*1*_* P*_*1*_	*t*_*2*_* P*_*2*_	*t*_*3*_* P*_*3*_
BCVA(logMAR)	0.873 ± 0.265	0.666 ± 0.176	0.512 ± 0.141	18.771, ≤ 0.001	3.506, 0.001	6.105 ≤ 0.001	2.599,0.012
CMT(μm)	476.183 ± 115.653	399.510 ± 53.979	245.826 ± 20.875	56.769, ≤ 0.001	3.482, 0.001	10.462, ≤ 0.001	6.980, ≤ 0.001
implicit time of N1 wave from ring 1(ms)	31.685 ± 2.799	28.729 ± 1.337	24.227 ± 1.300	86.023, ≤ 0.001	5.161, ≤ 0.001	13.024, ≤ 0.001	7.863, ≤ 0.001
implicit time of P1 wave from ring 1(ms)	50.523 ± 3.803	46.431 ± 3.349	43.587 ± 3.692	21.333, ≤ 0.001	3.833, ≤ 0.001	6.497, ≤ 0.001	2.664,0.010
amplitude density of P1 wave from ring 1 (nv/deg^2^)	15.809 ± 5.420	21.008 ± 4.704	27.417 ± 6.010	26.622, ≤ 0.001	3.262,0.002	7.284, ≤ 0.001	4.021, ≤ 0.001
implicit time of N1 wave from ring 2(ms)	29.888 ± 3.044	26.551 ± 2.981	22.547 ± 2.453	38.566, ≤ 0.001	3.987, ≤ 0.001	8.770, ≤ 0.001	4.784, ≤ 0.001
implicit time of P1 wave from ring 2(ms)	48.414 ± 3.684	44.670 ± 3.359	39.094 ± 2.767	46.681, ≤ 0.001	3.857, ≤ 0.001	9.601, ≤ 0.001	5.744, ≤ 0.001
amplitude density of P1 wave from ring 2(nv/deg^2^)	4.004 ± 1.467	6.676 ± 2.156	10.671 ± 2.006	71.764, ≤ 0.001	4.770, ≤ 0.001	11.902, ≤ 0.001	7.132, ≤ 0.001

*t*_*1*_* P*_*1*_ showed 1 W after treatment compared with baseline, *t*_*2*_* P*_*2*_ showed 2 M after treatment compared with baseline, *t*_*3*_* P*_*3*_ showed 2 M after treatment compared with 1 W after treatment

## Discussion

Long time edema can cause permanent damage, such as the loss of photoreceptor cells, the disorder of ellipsoid zone arrangement, the delay of P1 peak, and the decrease of amplitude, which are still irreversible after anti-VEGF treatment [4], which is related to the apoptosis caused by severe ischemia and hypoxia [5–7]. The purpose of this study is to explore the functional change of foveal and the correlation after structure improvement.

VEGF [8–10], as a vascular factor, not only plays an important role in regulating the generation of vascular endothelial cells but also has a protective effect on the central nervous system and retinal ganglion cells. Animal experiments show that the decrease of intracellular VEGF concentration will significantly reduce ganglion cells.Under the condition of mild ischemia caused by CRVO, the 30-Hz flicker response of full-field ERG will slightly increase [11, 12]. After intravitreal injection of bevacizumab, a series of changes occurred in mitochondria of photoreceptor inner segment cells [13], such as swelling, disordered arrangement, decreased density, loss of cristae, increase of amorphous corpuscles rich in phospholipids, and then apoptosis started.

Compared with monoclonal antibody drugs such as ranibizumab,conbercept is a novel anti-VEGF reagent, is a humanized soluble,VEGF receptor(VEGFR) protein comprising extra-cellular domain-2 of VEGFR-1, and extra-cellular domain-3 and-4 of VEGFR2. All of these domains are connected via the Fc region of human immunoglobulin G. The most notable characteristic of conbercept is that it binds not only VEGF-A, but also VEGF-B, VEGF-C, and placental growth factor(PIGF) all with high affinity [14]. Furthermore, the affinity of conbercept to VEGF-A is even greater than that of ranibizumab [15, 16]. After intravitreal conbercept, the fluid will gradually decrease to absorption, and the function of cells will be partially restored [9], the implicit time and amplitude will gradually become normal, and mf-ERG will be significantly improved.

This study showed that mf-ERG increased in a short time after intravitreal injection of conbercept, but it was not directly related to the best corrected visual acuity and the improvement of retinal thickness in fovea macular.The conclusion of this study was consistent with Rohit Shetty’s [17], which confirmed that conbercept had no toxic effect on cones and inner retinal cells in a short time.

The first and second rings of mf-ERG reflects the fovea and inner retinal function within 10°. The improvement of amplitude density and implicit time of P1 wave and N1 wave indicated that anti-VEGF could alleviate the ischemia and hypoxia condition of inner retina in a short time and break the positive feedback process of VEGF production.In this study, most patients suffered from photoreceptor cell loss and ellipsoid zone disorder before the treatment, and some patients had obvious changes in cell integrity and distribution two months after treatment, and the BCVA also improved significantly.

This study showed that the morphology and function of the macular of CRVO-ME patients improved significantly in a short time after anti-VEGF treatment and did not show the cytotoxic effect of anti-VEGF drugs, which was consistent with previous reports, but there was no correlation between them. There were several reasons for this: 1. The subretinal fluid of the foveal was decreased after anti-VEGF treatment, and the central visual acuity was improved. However the intensity of anti-VEGF treatment might not be enough, which could make the functions of cones and inner retinal cells not fully recovered, so there was no correlation between the improvement of macular function and structure. 2. Choriocaphillaris is the main source of blood supply to the macula, and its endothelial cells have loose junction, so oxygen is easy to diffuse to the inner layer cells. Whether conbercept would affect the permeability of its endothelial cells and reduce the intercellular space, thus reducing the fovea blood supply, have been rarely reported in the past. 3. Because of the small number of samples, short follow-up time, and the lack of preoperative evaluation of choroidal blood supply, the size, and the location of retinal capillary non-perfusion area, etc., whether these factors will affect the experimental results remains to be demonstrated in the next step.

## Conclusions

The BCVA, the structure, and the function of the macular were greatly improved after intravitreal conbercept for central retinal vein occlusion induced macular edema; however there was no significant correlation between the improvement of the function of macular with the structure of macular and vision.

## Data Availability

The datasets used and/or analysed during the current study are available from the corresponding author on reasonable request.
